# Papillary craniopharyngioma following an initial diagnosis of Rathke cleft cyst: a case report and narrative review

**DOI:** 10.1210/jcemcr/luag264

**Published:** 2026-09-11

**Authors:** Carmela Caputo, Penelope A McKelvie, Rana Dhillon, Paul D Smith

**Affiliations:** Department of Endocrinology and Diabetes, St Vincent's Hospital, Melbourne, Fitzroy, VIC 3065, Australia; Department of Medicine, The University of Melbourne, Melbourne, VIC 3010, Australia; Department of Medicine, The University of Melbourne, Melbourne, VIC 3010, Australia; Department of Anatomical Pathology, St Vincent's Hospital, Fitzroy, VIC 3065, Australia; Department of Medicine, The University of Melbourne, Melbourne, VIC 3010, Australia; Department of Neurosurgery, St Vincent's Hospital, Melbourne, Fitzroy, VIC 3065, Australia; Department of Medicine, The University of Melbourne, Melbourne, VIC 3010, Australia; Department of Neurosurgery, St Vincent's Hospital, Melbourne, Fitzroy, VIC 3065, Australia

**Keywords:** craniopharyngioma, papillary craniopharyngioma, Rathke cleft cyst

## Abstract

A 59-year-old man presented with a giant suprasellar cystic lesion causing obstructive hydrocephalus. Radiological and intraoperative findings suggested a cystic craniopharyngioma (CP); however, histopathology was interpreted as most consistent with a Rathke cleft cyst (RCC). Six years later, surveillance imaging demonstrated recurrence as a new mixed solid-cystic suprasellar mass with infundibular thickening. Histopathology showed papillary architecture with diffuse *BRAF V600E* immunopositivity, confirming papillary craniopharyngioma (PCP). The original specimen was subsequently re-examined, finding positivity for *BRAF V600E* staining and microscopic features supporting the diagnosis of PCP rather than RCC. We conducted a narrative review of published cases describing diagnostic overlap between RCC and CP. Several reports describe lesions with mixed histological features and cases initially labeled as RCC that were later reclassified as CP following additional sampling or modern molecular testing. Notably, nearly all such cases involved PCP rather than adamantinomatous CP. These observations suggest that some lesions initially diagnosed as RCC may represent under-recognized PCP, although a biological relationship between the 2 entities cannot be excluded. This case underscores the importance of integrating radiological, operative, and pathological findings when evaluating suprasellar cysts, additionally, the value of pathological reassessment—particularly *BRAF V600E* testing—when a presumed RCC demonstrates atypical features or interval progression.

## Introduction

Rathke cleft cysts (RCCs) and craniopharyngiomas (CPs) are uncommon epithelial lesions of the sellar and suprasellar region. In a review of 1356 abnormal pituitary magnetic resonance imaging (MRI) scans, 72.3% were pituitary adenomas, whereas only 3% were cystic lesions, of which RCCs were the most common, followed by CPs [1]. Although RCC and CP are traditionally regarded as distinct entities with different clinical, radiological, and histopathological features [2], their biological relationship remains incompletely understood. We describe a patient with an initially resected cystic lesion, histologically favored to be RCC, that recurred 6 years later as papillary craniopharyngioma (PCP). Retrospective histological analysis of the original specimen, utilizing *BRAF V600E* immunohistochemistry (IHC), confirmed the original lesion as a PCP. In the context of a narrative review of similar reports, this case highlights an uncommon but clinically relevant diagnostic overlap between RCC and CP, particularly PCP, while underscoring the possibility that some lesions initially diagnosed as RCC may represent under-recognized PCP because of sampling limitations or the absence of contemporary molecular testing.

## Case presentation

**Figure 1:**
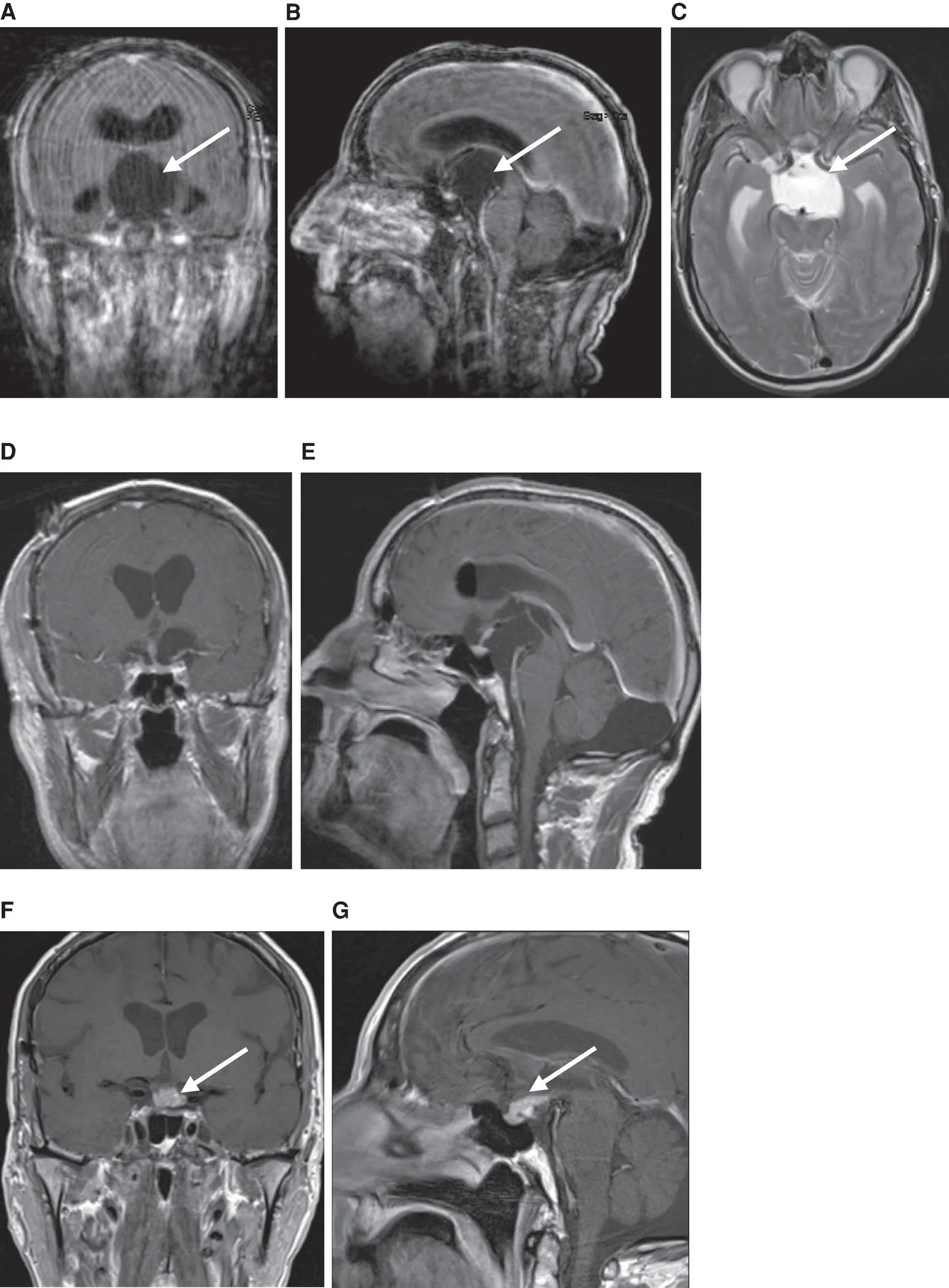
Magnetic resonance imaging (MRI) scans. A-C) At presentation in 2015: coronal post-contrast T1 (A), sagittal post-contrast T1 (B), and axial T2 (C) images demonstrating a large predominantly cystic suprasellar/interpeduncular lesion (white arrows) causing obstructive hydrocephalus with ventricular dilatation and periventricular edema. D-E) Postoperative imaging in 2015: coronal (D) and sagittal (E) post-contrast T1 images following right frontal craniotomy and cyst fenestration, demonstrating marked decompression of the cystic lesion and improvement in hydrocephalus. F-G) Recurrence in 2021: coronal (F) and sagittal (G) post-contrast T1 images demonstrating development of a new enhancing mixed solid-cystic suprasellar lesion adjacent to the pituitary stalk and optic chiasm (white arrows), later confirmed as papillary craniopharyngioma.

In 2015, a 59-year-old man presented with 10 months of cognitive disturbance, with family noting episodes of slurred speech and confusion. In the 3 days leading up to presentation he developed progressive headache and unsteadiness. An emergency MRI demonstrated marked hydrocephalus and a suprasellar cystic lesion with contents slightly hyperdense relative to cerebrospinal fluid (CSF) (Fig. 1A-1C). Due to the clinical features of raised intracranial pressure, he underwent urgent CSF diversion followed by right-sided craniotomy, partial cyst wall resection, and wide fenestration into the subarachnoid space. At surgery, the lesion contained pressurized fluid of milky appearance.

Histopathological assessment demonstrated a convoluted cyst wall with delicate fibrous stroma lined by flattened non-keratinizing stratified epithelium with squamous metaplasia. Cilia were not identified. Overall, the features were interpreted as a RCC; however, concern for PCP remained because of the extensive suprasellar location with associated hydrocephalus and the atypical intraoperative cyst contents.

Postoperatively, the patient improved markedly, with resolution of hydrocephalus and neurological symptoms. Available records did not demonstrate pituitary dysfunction. Serial MRI in 2016 and 2018 demonstrated a well-decompressed cyst cavity (Fig. 1D-1E). In 2021, at 65 years of age, surveillance MRI identified a new enhancing suprasellar lesion.

## Diagnostic assessment

The recurrence on the MRI scan demonstrated a 15 × 16 × 10 mm enhancing mixed solid-cystic suprasellar mass, with associated thickening of the infundibulum (Fig. 1F-1G). The mass was in contact with the optic chiasm, which was slightly effaced superiorly with posterior extension along the floor of the third ventricle and abutment of the mammillary bodies. This represented a change from the original cystic lesion with the development of a more solid-appearing component. At this time, the patient remained asymptomatic, with no visual field compromise, headache, nausea, vomiting, or evidence of pituitary insufficiency.

## Treatment

The patient proceeded to endoscopic transsphenoidal exploration of the lesion. At operation, the superior aspect of the pituitary gland was identified and protected. The narrow corridor between the optic chiasm superiorly and the pituitary gland inferiorly was entered, and the lesion was mobilized from the inferior surface of the optic chiasm. The stalk appeared displaced to the right, and the lesion seemed to arise from, or be closely adherent to, the stalk consistent with a CP. Further mobilization would have required division of or substantial manipulation of the stalk with a high risk of hypopituitarism; therefore, a conservative limited resection for diagnosis was performed.

**Figure 2:**
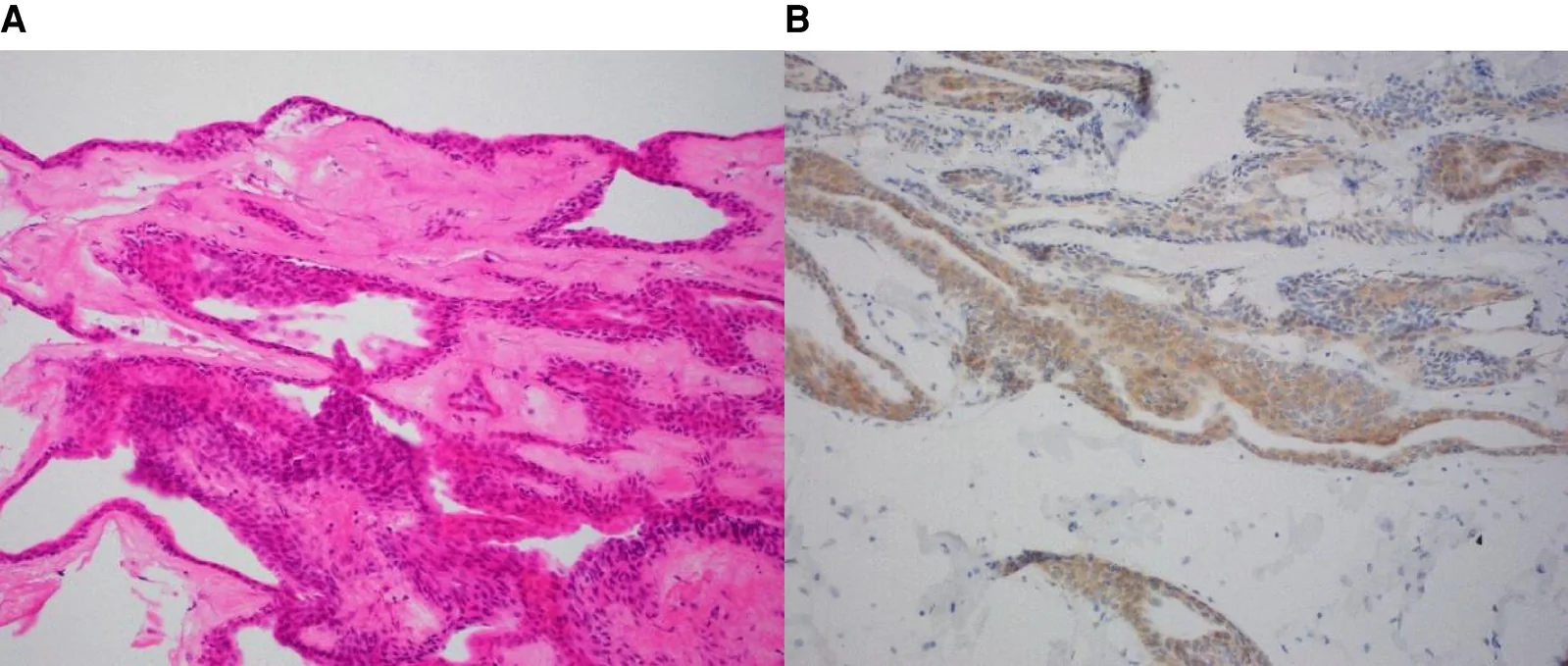
Histology and immunohistochemistry (IHC) of the lesion in 2021. A) Hematoxylin and eosin staining showing sheets of stratified squamous epithelium. with papillary architecture and focal inflammation. Magnification 20×. B) Positive *BRAF V600E* IHC. Magnification 20×.

**Figure 3:**
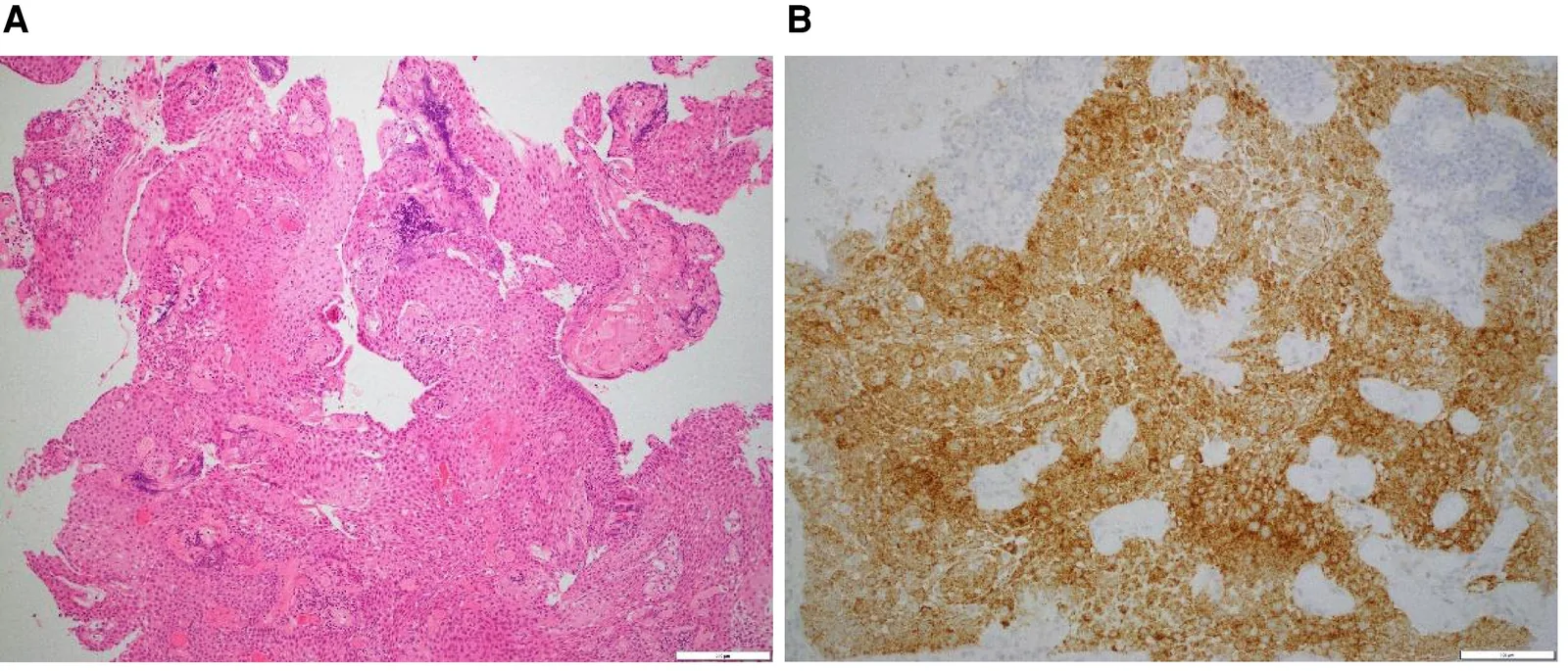
Retrospective histology and immunohistochemistry (IHC) of the original lesion in 2015. A) Hematoxylin and eosin staining shows papillary architecture of stratified squamous non-keratinizing epithelium. Magnification 200×. (B) Positive *BRAF V600E* IHC. Magnification 100×.

Histopathological examination demonstrated sheets of stratified squamous non-keratinizing epithelium with papillary architecture and focal inflammation. IHC showed strong diffuse positivity for *BRAF V600E*, confirming PCP. (Fig. 2A-2B). Beta-catenin showed membranous staining without nuclear accumulation, excluding an adamantinomatous CP (ACP). Considering these findings, the original sampling from 2015 was re-examined, finding papillary architecture of stratified squamous, non-keratinizing epithelium and strong diffuse positivity for *BRAF V600E* with IHC (Fig. 3A-3B).

## Outcome and follow-up

The patient recovered well after surgery with preserved pituitary function. He subsequently received adjuvant stereotactic radiotherapy of 54 Gray in 30 fractions. Follow-up MRI have shown progressive contraction of the enhancing component of the lesion.

## Discussion

RCCs and CPs are distinct epithelial lesions of the sellar and suprasellar region; however, differentiating them can be challenging when imaging and histology overlap [2-5]. Symptomatic RCCs commonly present with headache, visual disturbance, and pituitary dysfunction [6]. They are usually sellar or sellar-suprasellar, whereas purely suprasellar lesions are uncommon [7, 8]. Histologically, RCCs are classically lined by simple ciliated cuboidal or columnar epithelium with goblet cells. Squamous metaplasia (SM) may occur, but overt stratified squamous epithelium is not typical. Recurrent RCC requiring further surgery is uncommon, although SM metaplasia has been associated with recurrence risk [9, 10].

CPs comprise 2 histological subtypes: ACP and PCP [4]. ACP is more typical in childhood and is characterized by mixed solid-cystic morphology, calcification, wet keratin, and nuclear accumulation of beta-catenin [3, 11, 12]. In contrast, PCP occurs predominantly in adults and more often presents as a solid or mixed solid-cystic enhancing suprasellar lesion without calcification. Histologically, PCP is composed of well-differentiated stratified squamous epithelium forming papillary fronds and is strongly associated with *BRAF V600E* mutation (>95%), which has both diagnostic and therapeutic relevance [13, 14].

Our case demonstrated clinical, operative, and radiological features atypical for conventional RCC, including obstructive hydrocephalus, extensive suprasellar extension, and milky cyst contents at surgery. Although the original histological interpretation favored RCC, the atypical clinical picture prompted ongoing radiological surveillance. Six years later, the lesion recurred as a predominantly solid suprasellar mass with infundibular thickening with histology that was unequivocally consistent with PCP. Retrospective application of *BRAF V600E* IHC on the original specimen (which was not routinely available in 2015) showed strong diffuse positivity, and re-evaluation found papillary architecture of stratified squamous epithelium. These findings reclassified the lesion as a PCP from the outset.

To contextualize this case, we undertook a narrative review of the published literature using PubMed/MEDLINE from database inception to March 2026. Search terms included combinations of “*Rathke cleft cyst*,” “*Rathke's cleft cyst*,” “*Rathke pouch*,” AND “*craniopharyngioma*,” “*papillary craniopharyngioma*,” and “*adamantinomatous craniopharyngioma*,” and reference lists of relevant articles were screened for additional reports. We identified 11 reports describing coexistent RCC and CP within the same lesion (Table 1), 4 reports in which lesions initially diagnosed as RCC were retrospectively reclassified as CP following modern molecular or immunohistochemical analysis (Table 2), and 8 reports describing recurrence with subsequent diagnosis of CP after an initial diagnosis of RCC (Table 3). Earlier reports lacking contemporary *BRAF V600E* analysis were retained for completeness but interpreted cautiously. Although the available literature is limited to case reports and is therefore susceptible to publication and selection bias, several observations are noteworthy. Across the published cases summarized in Tables 1-3, RCC was overwhelmingly associated with PCP, with only a single reported case of ACP [24]. In a study by Hofmann et al, among 30 RCCs and 51 cystic CPs (42 ACP, 9 PCP), analysis of a subgroup of 12 purely cystic CPs identified a single instance in which RCC “coincided” with PCP, with no cases coinciding with ACP [3]. A coexistent RCC and PCP has also been reported in a single Wistar rat [31]. An additional observation across these reported cases (Tables 1-3) is an apparent male predominance, previously noted by Sato et al in 2006 [21]. However, the number of published cases is too small to infer biological significance.

**Table 1:** Published cases of coexisting Rathke cleft cyst and craniopharyngioma

First author, Year [Reference]	Case	Presentation	Histology	Classification status
Matsushima et al, 1980 [15]	48-year-old male	Polyuria, polydipsia, memory impairment, disorientation; large suprasellar mass compressing the third ventricle	Drainage of mucinous, pus-like material suggestive of mucocele; no further histologic detail available at first operation. Recurrence at 14 months: Two types of stratified epithelium identified, including papillary projections lined by stratified squamous epithelium with a superficial columnar cell layer and lower germinal cell layers; EM showed ciliated and non-ciliated secretory cells, including goblet cells	**RCC and PCP**
Tajika et al, 1982 [16]	11-year-old male	Visual disturbance; intrasellar cyst; gelatinous fluid	Ciliated columnar/cuboidal epithelium with goblet cells; focal stratified squamous epithelium with cholesterol clefts	**RCC and PCP**
Tajika et al, 1982 [16]	21-year-old female	Visual disturbance, headache, menstrual disturbance; suprasellar lesion with calcification	Ciliated columnar/cuboidal epithelium with goblet cells; focal stratified squamous epithelium with cholesterol clefts	**RCC and PCP**
Tajika et al, 1982 [16]	37-year-old male	Visual disturbance, headache; suprasellar lesion; yellow turbid fluid	Stratified squamous epithelium; CP with focal partially ciliated cuboidal/columnar epithelium	**RCC and PCP**
Tajika et al, 1982 [16]	28-year-old male	Visual disturbance, headache; suprasellar lesion; pus-filled cyst	Stratified squamous epithelium; CP with focal partially ciliated cuboidal/columnar epithelium	**RCC and PCP**
Nakajou et al, 1994 [17]	74-year-old female	Visual disturbance, hyperprolactinemia; cystic and solid components	Ciliated columnar, mucous, and basal cells; solid component with stratified squamous epithelium; transition zone present	**RCC and PCP**
Nakajou et al, 1994 [17]	12-year-old male	Polyuria/polydipsia, growth restriction; cystic and solid components	Ciliated columnar, mucous, and basal cells; solid component with stratified squamous epithelium; transition zone present	**RCC and PCP**
Goodrich et al, 1985 [18]	48-year-old male	Visual disturbance, headache, hormonal disturbance; cystic suprasellar lesion	Stratified squamous epithelium typical of PCP; focal ciliated cuboidal/columnar epithelium; keratinization, inflammation, calcium crystals	**RCC and PCP**
Oka et al, 1997 [19]	36-year-old male	Visual disturbance; suprasellar lesion; solid mass with small cysts	PCP with foci of ciliated columnar epithelium; EM showing ciliated epithelium with goblet cells	**RCC and PCP**
Nishioka et al, 2000 [20]	49-year-old female	Visual disturbance, headache, nausea; recurrence after CP resection	Stratified squamous epithelium; CP with focal partially ciliated cuboidal/columnar epithelium	**RCC and PCP**
Sato et al, 2006 [21]	74-year-old male	Visual disturbance; cystic sellar/suprasellar lesion with solid component	Ciliated columnar epithelium with focal SM; solid component PCP with focal ciliated columnar epithelium	**RCC and PCP**

Abbreviations: ACP, adamantinomatous craniopharyngioma; CP, craniopharyngioma; EM, electron microscopy; IHC, immunohistochemistry; PCP, papillary craniopharyngioma; RCC, Rathke cleft cyst; SM, squamous metaplasia.

**Table 2:** Published cases of Rathke cleft cyst reclassified as craniopharyngioma after retrospective analysis in a single lesion

First author, Year [Reference]	Case	Presentation	Histology	Classification status
Marucci et al, 2015 [22]	12-year-old male	Not reported	**Considered RCC** Small sheets of squamous epithelium with clefts and pseudopapillary architecture	**Retrospective PCP** Next-generation sequencing showed a *BRAF* exon 15 alteration
Schweizer et al, 2015 [14]	42-year-old male	Cystic sellar/suprasellar lesion with creamy contents	**Considered RCC** Compact squamous epithelium reminiscent of PCP; no β–catenin nuclear accumulation; no wet keratin or calcification	**Retrospective PCP** SM present, no cilia identified; *BRAF V600E* positive by IHC and pyrosequencing
Schweizer et al, 2015 [14]	30-year-old male	Cystic sellar/suprasellar lesion; intraoperative impression of CP	**Considered RCC** Squamous epithelium lining cyst wall; no β–catenin nuclear accumulation; no wet keratin or calcification	**Retrospective PCP** SM present, no cilia identified; *BRAF V600E* positive by IHC and pyrosequencing
Schlaffer et al, 2018 [23]	6-year-old (sex not reported)	Sellar/suprasellar lesion with RCC-like radiology; viscous yellow cyst contents	**Considered RCC** Apically ciliated columnar epithelium with goblet cells; well-differentiated squamous epithelium	**Retrospective PCP** *BRAF V600E* positive by IHC

Abbreviations: CP, craniopharyngioma; EM, electron microscopy; IHC, immunohistochemistry; PCP, papillary craniopharyngioma; RCC, Rathke cleft cyst.

**Table 3:** Cases initially diagnosed as Rathke cleft cyst and subsequently reclassified as craniopharyngioma following recurrences

First author, Year [Reference]	Case	Presentation	First histology	Recurrence	Reclassification
Park et al, 2009 [24]	41-year-old male	Visual disturbance; suprasellar cystic lesion; mucoid milky-white fluid obtained at biopsy	Ciliated columnar epithelium typical of RCC; no nuclear β-catenin accumulation	34 months	**ACP** Solid and cystic components with attenuated epithelium, palisaded basal layer, nuclear β-catenin accumulation, and wet keratin
Okada et al, 2010 [25]	61-year-old male	Visual disturbance; intrasellar cystic lesion; turbid whitish fluid encountered at surgery	Ciliated columnar epithelium with multilayered basal cells; basal cell hyperplasia and SM	3 months; recurrence with larger multilobulated mural nodules	**PCP** Stratified squamous epithelium in a papillary arrangement, with focal cilia
Ogawa et al, 2014 [26]	47-year-old male	Visual disturbance; 21 mm suprasellar cystic lesion; clear homogeneous mucus within a thin glittering inner wall, with minor cerebrospinal fluid leakage; second surgery at 1 month	Samples from the first and second surgeries showed single-layered ciliated columnar epithelium with goblet cells and focal squamous epithelium, consistent with RCC	6 months	**PCP** stratified squamous epithelium in a papillary arrangement; β-catenin membranous only, without nuclear translocation; Ki-67 index: 20%. Suspected malignant potential
Schweizer et al, 2015 [14]	76-year-old male	Surgery 14 years earlier, initially considered RCC; no further clinical detail available	Specimen unavailable	14 years	**PCP** No nuclear β-catenin accumulation; *BRAF V600E* positive by IHC
Manjila et al, 2019 [27]	46-year-old male	Visual disturbance; sellar/suprasellar cystic mass with solid components	Blood, necrotic debris, and scant cuboidal epithelial fragments; differential diagnosis included necrotic pituitary adenoma, ruptured RCC, or both	1 month	**PCP with RCC** Ciliated cuboidal lining identified
Sharma et al, 2024 [28]	39-year-old female	Visual disturbance and menstrual irregularity; MRI showed a sellar/suprasellar cystic lesion with a solid component; yellow fluid encountered at surgery	Stratified columnar epithelium with SM; no nuclear β-catenin accumulation	3 months; recurrent lesion became more lobulated and cystic	**PCP** No nuclear β-catenin accumulation; *BRAF V600E* positive by IHC
Mattheisen et al, 2025 [29]	74-year-old male	MRI showed a suprasellar cystic lesion compressing the optic chiasm and extending into the third ventricle	Squamous epithelium with SM; no wet keratin or calcification; negative IHC for β-catenin and BRAF V600E; interpreted as RCC with SM	4 months	**PCP** *BRAF V600E* positive by IHC and pyrosequencing
Candy et al, 2025 [30]	63-year-old male	Dizziness; incidental 25 × 30 mm simple sellar/suprasellar cyst compressing the optic chiasm	Histopathology initially consistent with RCC; second recurrence also interpreted as RCC, with BRAF V600E negative by IHC	Time to first recurrence not reported; 3 further recurrences	**PCP** *BRAF V600E* positive by IHC and next-generation sequencing at the third recurrence

Abbreviations: ACP, adamantinomatous craniopharyngioma; CP, craniopharyngioma; EM, electron microscopy; IHC, immunohistochemistry; MRI, magnetic resonance imaging; PCP, papillary craniopharyngioma; RCC, Rathke cleft cyst; SM, squamous metaplasia.

Several hypotheses have been proposed to explain an observed relationship between RCC and PCP. One possibility is that some lesions represent progression from RCC to PCP [26, 32], another is that both lesions arise from a shared embryological precursor within Rathke pouch remnants which subsequently diverge along distinct epithelial pathways [15, 18]; however, direct evidence for either transformation or a common progenitor remains lacking. Jakob Erdheim—regarded as the father of CP pathology—first described squamous epithelial rests derived from remnants of the craniopharyngeal duct on the dorsal surface of the anterior pituitary extending into the pars tuberalis and proposed that these rests may serve as precursors of CP [33]. Squamous epithelial rests along the pars tuberalis have subsequently been identified in fetal, infant, and adult pituitary specimens, supporting the persistence of such remnants from early development [34, 35]. It may be plausible that incorporation of these epithelial rests into Rathke pouch–derived cysts may explain the rare cases of coexistence of RCC and PCP, although this remains speculative.

A central interpretive challenge in cases such as this is determining whether the observed sequence represents true biological evolution, coexistence of 2 epithelial processes, or is due to sampling limitations inherent to cystic lesions. In our case, the unequivocal finding of PCP at recurrence, together with retrospective re-analysis of the original pathology using *BRAF V600E* IHC, supports sampling limitations as the most likely explanation.

Our case highlights the difficulty of differentiating RCC and CP histologically, and the value of considering *BRAF V600E* mutation testing. It illustrates that an initial pathological impression of RCC may not fully exclude PCP when the clinical, operative, or radiological features are atypical. However, a small number of cases in the literature show that RCC and CP histological features can coexist, and that when a CP component is present, it is overwhelmingly a PCP rather than an ACP. This recurring pattern across multiple reports makes a purely coincidental association unlikely. Until the basis of this association is better understood, clinicians should integrate radiological, operative, histological, and molecular findings, and consider repeat pathological review and molecular testing when a presumed RCC demonstrates atypical features or interval change in behavior.

## Learning points

- Rathke cleft cyst and craniopharyngioma are distinct epithelial lesions; however, a small number of reports have described coexistent lesions, especially involving papillary craniopharyngioma.
- With the advent of *BRAF V600E* testing, a number of cases in the literature initially diagnosed as Rathke cleft cyst have been reclassified as craniopharyngiomas.
- An initial histological impression of Rathke cleft cyst in an atypical cystic lesion does not fully exclude craniopharyngioma, particularly when operative or radiological features are discordant with a simple Rathke cleft cyst.
- When clinical, operative, or radiological features are atypical or change over time, repeat pathological review and *BRAF V600E* testing should be considered, particularly in lesions previously diagnosed as Rathke cleft cyst.

## Contributors

All authors made substantive contributions to authorship. C.C. conceived the report and drafted the manuscript. P.Mc.K., R.D., and P.D.S. were involved in the diagnosis and management of the patient. All authors were involved with critically reviewing, editing for important intellectual content, and approving the final draft and are accountable for all aspects of the work.

## Data Availability

All data generated or analyzed during this study are included in this published article.

## Abbreviations

ACP: adamantinomatous craniopharyngioma
CP: craniopharyngioma
IHC: immunohistochemistry
MRI: magnetic resonance imaging
PCP: papillary craniopharyngioma
RCC: Rathke cleft cyst
